# EYA4 promotes cellular senescence by enhancing *P21* transcription through interaction with SIX2

**DOI:** 10.1007/s44307-026-00109-8

**Published:** 2026-04-17

**Authors:** Xiaolin Li, Pingsu Mao, Dandan Chen, Lin Li, Hehua Fang, Junjiu Huang, Haiying Liu

**Affiliations:** 1 MOE Key Laboratory of Gene Function and Regulation, State Key Laboratory of Biocontrol, Guangdong Key Laboratory of Pharmaceutical Functional Genes, School of Life Sciences, Sun Yat-Sen University, Guangzhou, Guangdong 510275 People’s Republic of China; 2 Institute for Cancer Medicine, School of Basic Medical Sciences, Southwest Medical University, Luzhou, 646000 People’s Republic of China

**Keywords:** EYA4, SIX2, P21, Cellular senescence, P53

## Abstract

During the aging process, the expression levels of numerous genes undergo significant changes, some of which in turn regulate the progression of aging. In this study, we identified the expression of EYA4 is upregulated during aging and demonstrated its critical role in modulating cellular senescence. Knockdown of *EYA4* significantly delays both replicative and stress-induced cellular senescence. Mechanistic investigations showed that EYA4 interacts with the transcription factor SIX2 to promote the expression of p21, a key molecule in the senescence-signaling pathway, which accelerates cellular senescence. Interestingly, EYA4 possesses both transcriptional activation and phosphatase activities, yet experiments using phosphatase-deficient mutants revealed that its ability to enhance p21 expression is independent of its phosphatase activity. Further analysis demonstrated that the EYA4-SIX2-mediated regulation of p21 expression is p53-dependent, as the absence of p53 abolished this regulatory effect. In conclusion, our findings uncover a novel role of the EYA4-SIX2 complex in promoting cellular senescence through the transcriptional activation of p21. Targeting EYA4 may represent a promising strategy for delaying the aging process.

**Supplementary Information:**

The online version contains supplementary material available at 10.1007/s44307-026-00109-8.

## Introduction

Cellular senescence is widely accepted as a state of irreversible cell cycle arrest and is triggered by multiple stimuli, including but not limited to replicative stress, telomere shortening, reactive oxygen species (ROS), DNA damage and oncogene activation (Sturmlechner et al. 2017). It plays a dual role in promoting tissue repair and tumor suppression, while also contributing to tissue dysfunction and the progression of chronic diseases when improperly regulated (Demaria et al. 2014; Kennedy et al. 2014; Muñoz-Espín et al. 2013; Storer et al. 2013). Investigating the molecular mechanisms underlying cellular senescence provides insights into the broader context of aging and holds potential for developing therapeutic strategies to mitigate age-associated pathologies.

Among the key regulators of cellular senescence, the cyclin-dependent kinase inhibitor p21 (encoded by the *CDKN1A* gene, or *P21*) plays a pivotal role. As a downstream effector of the p53 (encoded by the *TP53* gene, or *P53*) pathway, p21 orchestrates cell cycle arrest in response to DNA damage and stress signals, thereby acting as a safeguard against uncontrolled proliferation (Abbas and Dutta 2009; Kastenhuber and Lowe 2017). However, its overexpression is often implicated in the onset of cellular senescence. Understanding how p21 expression is regulated in senescence contexts is therefore vital for both basic research and clinical applications.

EYA4 (Eyes Absent Homolog 4), a member of the EYA family of proteins, has been extensively studied for its involvement in transcriptional regulation, DNA damage repair, and tissue development (Xu et al. 1999). It is characterized by a conserved C-terminal EYA domain (ED) with tyrosine phosphatase activity and protein interaction functions, as well as an N-terminal domain (NTD) with threonine phosphatase activity and transcriptional co-activation functions (Ikeda et al. 2002; Okabe et al. 2009; Tootle et al. 2003; Xu et al. 1997). Emerging studies suggest that EYA4 is not only crucial for maintaining cellular homeostasis but also plays a role in pathological processes such as cancer and hearing loss (el-Deiry et al. 1993; Ohto et al. 1999; Okabe et al. 2009; Rayapureddi et al. 2003). However, its function in regulating cellular senescence remains largely unexplored.

In this study, we observed an age-related increase in EYA4 expression. To investigate its potential role, we explored how EYA4 contributes to cellular senescence and uncovered the underlying mechanisms driving this process.

## Methods

### Analysis of EYA4 expression levels using GTEx data

Transcriptome files and donor information were downloaded from the GTEx website (https://www.gtexportal.org/home/datasets) ("The Genotype-Tissue Expression (GTEx) project," 2013). Normalized TPM values were used to examine alterations in *EYA4* expression during aging. Participants were categorized into two groups: young (ages 20–49) and old (ages ≥ 60). TPM values were compared between the two groups using Student’s *t*-test (* *p* < 0.05, ** *p* < 0.01, *** *p* < 0.001).

### Cell culture

HEK293T (ATCC Cat# CRL-3216, RRID: CVCL_0063), HeLa (ATCC Cat# CCL-2, RRID: CVCL_0030), HFF-1 (ATCC Cat# SCRC-1041, RRID: CVCL_3285), and BJ (ATCC Cat# CRL-2522, RRID: CVCL_3653) cells were obtained from the American Type Culture Collection (ATCC, Manassas, VA, USA). To get replicative senescent cells, HFF-1 and BJ cells were cultured for more than 50PD, whereas the young cells were about 25PD. To get stress-induced senescent cells, young HFF-1/BJ cells (~ 25PD) or HeLa cells were treated with bleomycin (BLM, 2.5 μM) or saline (Ctl) for 3 days. All cells were cultured in Dulbecco’s modified Eagle’s medium (DMEM, GIBCO, Thermo Fisher Scientific, Waltham, MA, USA) with 10% fetal bovine serum (FBS, GIBCO) and 1% penicillin/streptomycin (GIBCO) at 37 °C and 5% CO_2_. All cells have no mycoplasma contamination in this study.

### Plasmids and transfection

Human wild type *EYA4*, *SIX2*, *P53* genes were amplified from HEK293T cDNAs and cloned into pLVX-GFP or pLenti-HA/Flag plasmids. *EYA4* phosphatase mutations were generated by site-directed mutation based on wild-type *EYA4* plasmids. *EYA4* truncated plasmids were generated with the template of wild-type *EYA4* plasmids. Plasmids were transfected into HEK293T and HeLa cells using Polyethylenimine (PEI, Thermo Fisher Scientific) and FuGENE HD (Promega, Madison, WI, USA) transfection reagents respectively. siRNAs were transfected into HeLa cells by LipofectamineRNAiMAX (Thermo Fisher Scientific) transfection reagent. Sequences of the siRNAs are si*EYA4*−1, 5’-GAGUGGACUUUCCCAAACUTT-3’; si*EYA4*−2, 5’-GGAGCGUAUAUGACAUC-GAT-T-3’; si*SIX2*−1, 5’-AGAAUGAAAGCGUGCUCAATT-3’; si*SIX2*−2, 5’-GCGAG-CACCUUCACAAGAATT-3’; siNC, 5’-UUCUCCGAACGUGUCACGUTT-3’. For depletion of *P53* gene, the *P53* sgRNA (5’-CACCGCCATTGTTCAATATCGTCCG-3’) was cloned into lenti-CRISPR v2 plasmids. Lentiviruses were packaged in HEK293T cells by PEI transfection reagent and were infected into HeLa cells. Stable *P53-KO* HeLa cells were selected by 2 μg/mL puromycin. Scrambled sequence was used as negative control.

### Immunoblotting

Cells were harvested and lysed by SDS loading buffer in boiling water for 10 min. Proteins were separated by SDS-PAGE and transferred to PVDF membrane. The following primary antibodies were used in this study: anti-EYA4 (Santa Cruz Biotechnology Cat# sc-393111), anti-SIX2 (Proteintech Cat# 66347–1-Ig, RRID: AB_2881727), anti-p53 (Santa Cruz Biotechnology Cat# sc-126, RRID: AB_628082), anti-p21 (Cell Signaling Technology Cat# 2947, RRID: AB_823586),

anti-α-tubulin (Proteintech Cat# 66031–1-Ig, RRID: AB_11042766), anti-GAPDH (Proteintech Cat# 60,004–1-Ig, RRID: AB_2107436), anti-β-actin (Proteintech Cat# 60,008–1-Ig, RRID: AB_2289225), anti-GFP (Sangon Biotech Cat# D110008, RRID: AB_3697475), and anti-Flag (Sigma-Aldrich Cat# F1804, RRID: AB_262044). The secondary antibodies were HRP-conjugated anti-rabbit or anti-mouse antibodies (KPL, Gaithersburg, MD, USA). For protein half-life assay, HeLa cells were treated with 100 μg/mL cycloheximide (CHX) for the indicated times after plasmid transfection.

### Immunofluorescence (IF) and EdU staining

Cells were grown on coverslips and fixed with freshly prepared 4% paraformaldehyde for 15 min. The fixed cells were permeabilized with 0.2% Triton X-100 for 30 min, and then blocked with 5% goat serum for 1 h. The primary antibodies and fluorescence-labeled secondary antibodies were sequentially incubated with the fixed cells overnight at 4 °C or for 1 h at room temperature, respectively. Then cells were stained with DAPI (Vector Labs, Burlingame, CA, USA) and visualized using a fluorescence microscope. For EdU staining, cells were treated with 100 μM EdU for 30 min before fixation. Permeabilized cells were stained for 10 min at room temperature in staining buffer (1 mM CuSO4, 10 μM FAM-azide, 10 mM L-ascorbic acid in PBS). After washing with PBST, EdU-positive cells were visualized using a fluorescence microscope.

### Co-immunoprecipitation (Co-IP)

HEK293T or *P53-KO* HeLa cells were lysed in RIPA buffer (1% NP-40, 0.25% sodium deoxycholate, 50 mM Tris–HCl, 150 mM NaCl, 1 mM EDTA, 1 mM PMSF) containing phosphatase and/or proteinase inhibitors (Beyotime Biotechnology, Shanghai, China). GFP-tagged or Flag-tagged proteins were immunoprecipitated overnight at 4 °C using GFP-beads or Flag-beads (AlpalifeBio, Shenzhen, China) respectively. After washing, precipitated proteins were separated by SDS-PAGE followed by immunoblotting.

### Quantitative real-time PCR (RT-qPCR)

Total RNA was extracted using RNAiso Plus Reagent (9109, TaKaRa, Tokyo, Japan). 1 μg of total RNA was reverse-transcribed into cDNA using PrimeScript RT reagent Kit (RR047, TaKaRa). Target genes were amplified using RealStar Fast SYBR qPCR Mix (A301-10, GenStar, Beijing, China) with primers listed in Table S1. *GAPDH* was used as internal control. Data were analyzed using the comparative Ct (2^−ΔΔCt^) method.

### Chromatin immunoprecipitation (ChIP) and qPCR

HeLa cells were cross-linked for 10 min at room temperature by adding formaldehyde to the medium to a final concentration of 1%. The cross-linking was terminated by adding glycine to a final concentration of 1.25 mM for 5 min. After washing twice with ice-cold PBS containing 1 mM PMSF, cells were scraped and collected into a centrifuge tube, and then were pelleted at 1500 rpm for 5 min at 4 °C. The pellets were lysed using 200 μL ice-cold lysis buffer (1% SDS, 10 mM EDTA, 50 mM Tris–HCl) containing 1 mM PMSF and proteinase inhibitors for 10 min on ice, followed by sonication to generate DNA fragments averaging 500 bp. Samples were centrifuged at 12000 rpm at 4 °C for 10 min, and the supernatants were transferred to new tubes and diluted 10 times with dilution buffer (0.01% SDS, 1.1% Triton X-100, 1.2 mM EDTA, 16.7 mM Tris–HCl, 167 mM NaCl). The diluted samples were incubated with GFP-beads overnight at 4 °C to enrich DNA. The beads were then washed twice with low salt wash buffer (1% Triton X-100, 2 mM EDTA, 0.1% SDS, 150 mM NaCl, 20 mM Tris–HCl), twice with high salt wash buffer (1% Triton X-100, 2 mM EDTA, 0.1% SDS, 500 mM NaCl, 20 mM Tris–HCl), once with LiCl buffer (0.25 M LiCl, 1% NP-40, 1% sodium deoxycholate, 1 mM EDTA, 10 mM Tris–HCl), and twice with TE buffer (10 mM Tris–HCl, 1 mM EDTA). DNA was eluted from the beads twice using 250 μL elution buffer (1% SDS, 100 mM NaHCO_3_) at room temperature for 15 min each time. DNA–protein cross-linking was reversed by adding NaCl to a final concentration of 0.2 M for 4 h at 65 °C, followed by protein digestion with proteinase K for 1.5 h at 55 °C. DNA was extracted by conventional phenol/chloroform purification and then analyzed by qPCR. Primers were listed in Table S2.

### SA-β-gal staining

The SA-β-gal (senescence-associated beta-galactosidase) staining assay was performed with an SA-β-gal staining kit (Beyotime Biotechnology) according to the manufacturer’s instructions.

### Dual-luciferase reporter assay

The *P21* promoter sequences were constructed into pGL4.10 plasmid containing a firefly luciferase reporter gene. The pGL4.10 plasmids and renilla luciferase reporter gene-containing plasmids were transfected into cells which were plated in a 96-well plate. Luciferase activity was measured by dual-luciferase reporter assay system kit (Promega) according to the manufacturer’s instructions.

### Gene expression analysis of SIX family

To analyze the expression correlation between EYA4 and SIX family members, we employed the Gene Expression Profiling Interactive Analysis (GEPIA) database (http://gepia.cancer-pku.cn/) using the Genotype-Tissue Expression (GTEx) dataset, with all tissue types. The correlation was determined using the Pearson method based on expression data (TPM value).

To analyze the expression level differences between the SIX family members, the TPM values were downloaded from the GTEx database, and unpaired Student’s two-tailed t-test was used to determine the statistical significance between SIX1, SIX2, and SIX4.

### Statistics

All experiments were performed with at least three independent replicates. The sample sizes for the GTEx dataset are indicated in the corresponding figure legends. The statistical analysis was performed with GraphPad 8. All results were shown as mean ± SEM. The unpaired Student’s two-tailed t-test was used to determine the statistical significance (ns *p* > 0.05, * *p* < 0.05, ** *p* < 0.01, *** *p* < 0.001, **** *p* < 0.0001).

## Results

### EYA4 is upregulated in aging tissues and senescent cells

**Fig. 1:**
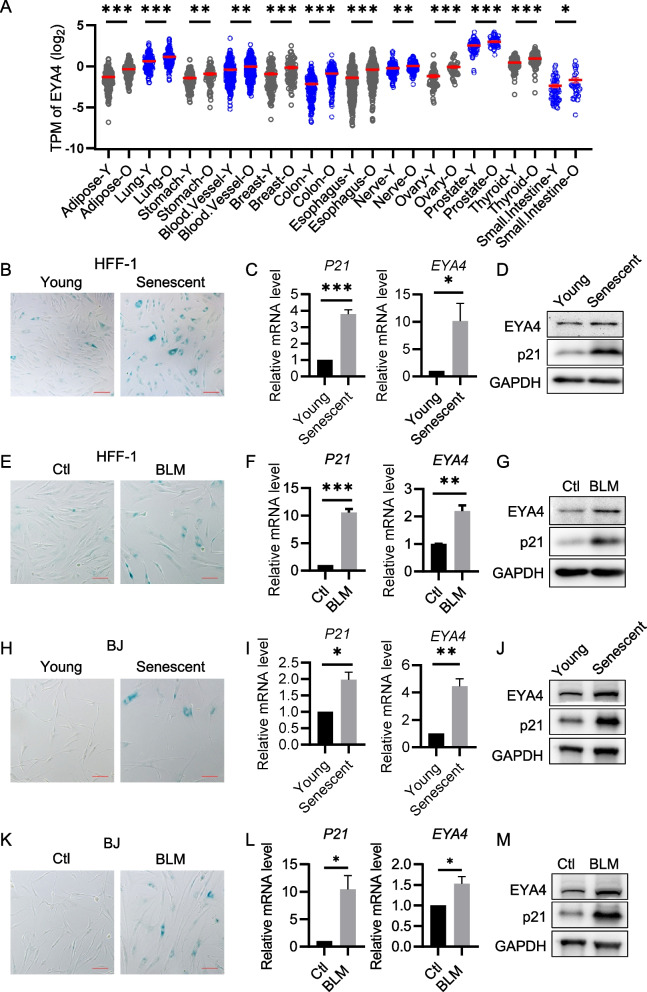
EYA4 is upregulated in senescent primary human diploid fibroblasts.** A** Expression levels of EYA4 in 12 tissues from young (20 ~ 49 years) and elderly (≥ 60 years) individuals based on RNA-seq data obtained from the GTEx database. The sample size was between 31 and 384 across tissues. Mean values ± SEM were shown. The y-axis was log₂-transformed to improve visualization of differences across a wide range. **B** SA-β-gal staining of replicative senescent (PD > 50) or control (~ 25PD) HFF-1 cells. **C** RT-qPCR analysis of *P21* and *EYA4* in HFF-1 cells described in (**B**). **D** Immunoblot analysis of p21 and EYA4 in HFF-1 cells described in (**B**). **E–G** Same as **B**–**D**, respectively, for stress-induced senescent HFF-1 cells. To induce cellular senescence, HFF-1 cells (~ 25 PD) were treated by bleomycin (BLM, 2.5 μM) or saline (Ctl) for 3 days. **H**–**M** Same as (**B**–**G**), respectively, for BJ fibroblasts under the same conditions. Scale bars, 100 μm. All values are means ± SEM of three independent experiments. Two-tailed unpaired Student’s t-test was used to determine the statistical significance (* *p* < 0.05, ** p < 0.01, *** p < 0.001)

To investigate whether EYA4 is associated with aging, we first examined changes in *EYA4* gene expression during the aging process. Using RNA-seq data from the GTEx database ("The Genotype-Tissue Expression (GTEx) project," 2013), we analyzed EYA4 expression levels across 25 tissues in young (ages 20–49) and elderly (ages ≥ 60) individuals. The results revealed that EYA4 expression was significantly upregulated in 12 aging tissues (Fig. 1A), and not changed in other 13 tissues (Supplementary Figure S1). Next, we established cellular aging models using human diploid fibroblasts. Replicative senescent HFF-1 cells (PD > 50) were generated through continuous passaging, which showed higher SA-β-gal expression (Fig. 1B) and increased p21 expression (Fig. 1C-D). In the replicative senescent HFF-1 cells, both mRNA and protein levels of *EYA4* were upregulated (Fig. 1C-D). Next, stress-induced senescent HFF-1 cells were generated by BLM treatment, showing increased SA-β-gal activity and p21 expression (Fig. 1E–G). Similarly, EYA4 expression was also elevated in stress-induced senescent HFF-1 cells (Fig. 1F-G). We repeated these experiments in BJ fibroblasts and observed similar results (Fig. 1H-M). These findings reveal that EYA4 expression is elevated in both aging individuals and senescent cells, suggesting that EYA4 may play a crucial role in the aging process.

### EYA4 deficiency alleviates cellular senescence and increases cell proliferation

**Fig. 2:**
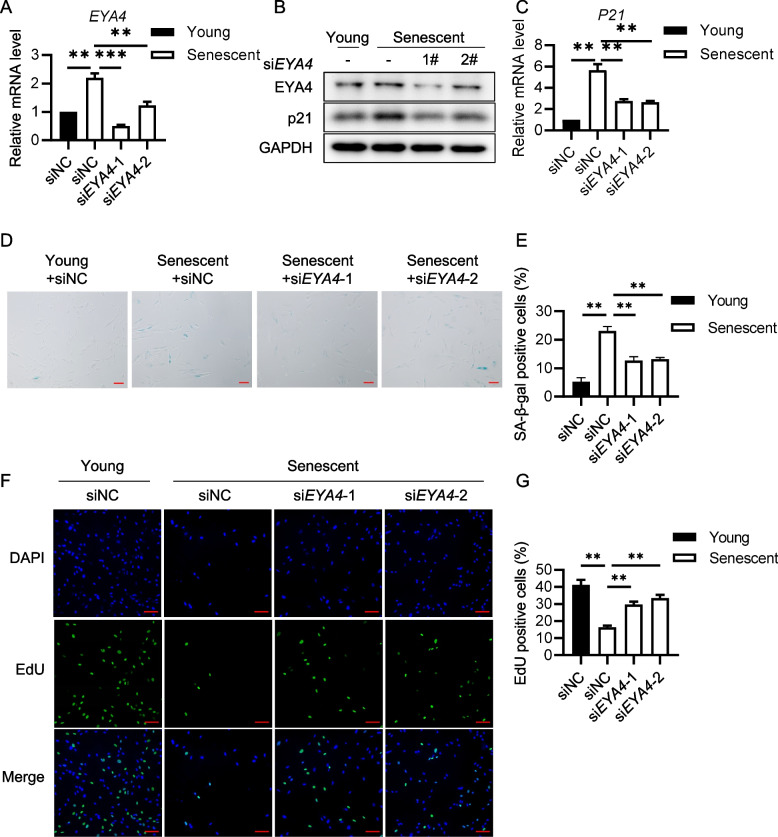
EYA4 deficiency alleviates replicative cellular senescence in primary human diploid fibroblasts. **A** *EYA4* knockdown efficiency. Young (~ 25PD) and pre-replicative senescent (~ 45PD) HFF-1 cells were transfected with scramble siRNA (NC) or *siEYA4*, and RT-qPCR was performed to detect knockdown efficiency 72 h later.** B** Immunoblot analysis of p21 and EYA4 in cells described in (**A**). **C**RT-qPCR analysis of *P21* in cells described in (**A**).** D** SA-β-gal staining to assess senescence in cells described in (**A**). **E** Quantification of (**D**). The percentage of SA-β-gal positive cells was calculated (n ≥ 100 × three repeats). **F** EdU incorporation assay of cells described in (A). Scale bars, 100 μm. **G** Quantification of (**F**). The percentage of EdU positive cells was calculated (n ≥ 100 × three repeats). All values are means ± SEM of three independent experiments. Two-tailed unpaired Student’s t-test was used to determine the statistical significance (* *p* < 0.05, ** p < 0.01, *** p < 0.001)

To investigate the role of EYA4 in cellular senescence, we knocked down *EYA4* expression in HFF-1 cells approaching replicative senescence (Fig. 2A-B) and continued culturing them to monitor the progression of senescence. The results showed that knockdown of *EYA4* reduced the levels of senescence markers, including p21 and SA-β-gal positivity (Fig. 2B-E). Senescent fibroblasts lose their ability to proliferate, while EdU incorporation into replicating DNA reflects cell proliferation capacity. Using EdU assay, we observed that cells with *EYA4* knockdown displayed significantly stronger EdU signals compared to the control group (Fig. 2F-G), indicating that many cells retained their ability to divide after *EYA4* depletion. These findings suggest that knocking down *EYA4* slows down the progression of replicative senescence.

**Fig. 3:**
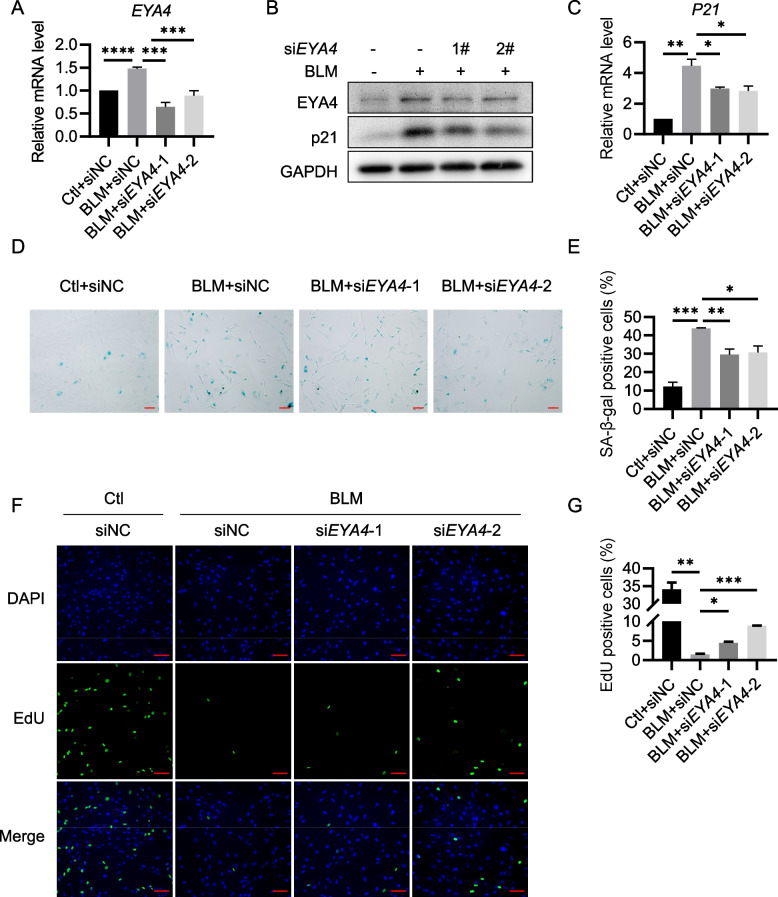
EYA4 deficiency alleviates stress-induced senescence in primary human diploid fibroblasts.** A** *EYA4* knockdown efficiency. HFF-1 cells (~ 25PD) were transfected with scramble siRNA (NC) or *siEYA4*, and then treated by bleomycin (BLM, 2.5 μM) or saline (Ctl) for 3 days after siRNA transfection. RT-qPCR was performed to detect knockdown efficiency. **B** Immunoblot analysis of p21 and EYA4 in cells described in (**A**). **C** RT-qPCR of *P21* in cells described in (**A**). **D** SA-β-gal staining to assess senescence in cells described in (**A**). **E** Quantification of (**D**). The percentage of SA-β-gal positive cells was calculated (n ≥ 100 × three repeats). **F** EdU incorporation assay of cells described in (**A**). Scale bars, 100 μm.** G** Quantification of (**F**). The percentage of EdU positive cells was calculated (n ≥ 100 × three repeats). All values are means ± SEM of three independent experiments. Two-tailed unpaired Student’s t-test was used to determine the statistical significance (* *p* < 0.05, ** p < 0.01, *** p < 0.001, **** p < 0.0001)

We repeated the experiments above in bleomycin-induced senescent HFF-1 cells (Fig. 3A-B) and found that knocking down *EYA4* reduced senescence markers, including p21 and SA-β-gal (Fig. 3B-E), and more cells retained their proliferative capacity (Fig. 3F-G). These results suggest that knocking down *EYA4* can also delay stress-induced cellular senescence.

### EYA4 enhances p21 expression independent of its phosphatase activity

**Fig. 4:**
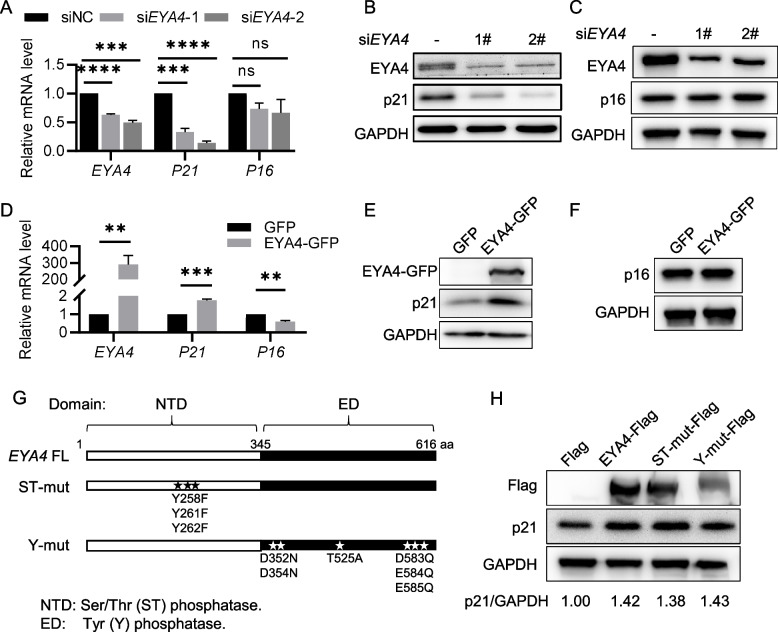
EYA4 regulates p21 expression independent of its phosphatase activity. **A** RT-qPCR analysis of *EYA4*, *P21* and *P16* in *EYA4* knockdown HeLa cells. HeLa cells were transfected with *siEYA4* or siNC for 72 h and subjected to RT-qPCR. **B**, **C** Immunoblot analysis of EYA4, p21 (**B**) and p16 (**C**) expression levels in HeLa cells described in (**A**). **D** RT-qPCR analysis of *EYA4*, *P21* and *P16* in EYA4 overexpression HeLa cells. HeLa cells were transfected with *EYA4* plasmids for 48 h and subjected to RT-qPCR. The y-axis is truncated to omit intermediate values due to the high fold change of EYA4 overexpression. **E**,** F** Immunoblot analysis of p21 (**E**) and p16 (**F**) expression levels in EYA4 overexpression HeLa cells. **G** Schematic representation of wild-type EYA4 and its phosphatase-deficient mutants.** H** Immunoblot analysis of p21, wild-type EYA4 and phosphatase-deficient EYA4 expression levels in HeLa cells. HeLa cells were transfected with wild-type *EYA4* or phosphatase-deficient *EYA4* mutants for 48 h and subjected to immunoblot analysis. All values are means ± SEM of three independent experiments. Two-tailed unpaired Student’s t-test was used to determine the statistical significance (* *p* < 0.05, ** p < 0.01, *** p < 0.001, **** p < 0.0001)

The p53-p21 and p16-pRB pathways are two classic signaling pathways that initiate cellular senescence, with p21 and p16 commonly recognized as biomarkers of cell senescence (Khosla and Farr 2020). However, we observed that knockdown of *EYA4* resulted in a decrease in p21 expression, while p16 levels remained unaffected (Fig. 4A-C). Consistently, overexpression of EYA4 significantly upregulated p21 expression, whereas *P16* mRNA levels showed a slight decrease with no changes at the protein level (Fig. 4D-F). These findings suggest that the regulation of p21 expression by EYA4 may go beyond its role as a senescence marker and could involve direct modulation by EYA4.

EYA4 possesses both transcriptional activation and phosphatase functions, with its serine/threonine (Ser/Thr) phosphatase activity localized in the N-terminal domain (NTD) and tyrosine (Tyr) phosphatase activity in the Eya domain (ED) (Okabe et al. 2009; Rayapureddi et al. 2003). To investigate which function of EYA4 contributes to p21 regulation, we constructed *EYA4* phosphatase activity inactive mutants (Fig. 4G). Overexpression of either wild-type *EYA4* or the mutants revealed that both Ser/Thr phosphatase inactive mutant and Tyr phosphatase inactive mutant promoted p21 expression to the same extent as the wild-type *EYA4* (Fig. 4H). This indicates that EYA4 promotes p21 expression independent of its phosphatase activity, likely through its transcriptional activation function.

### EYA4 interacts with SIX2 to promote *P21* transcription

**Fig. 5:**
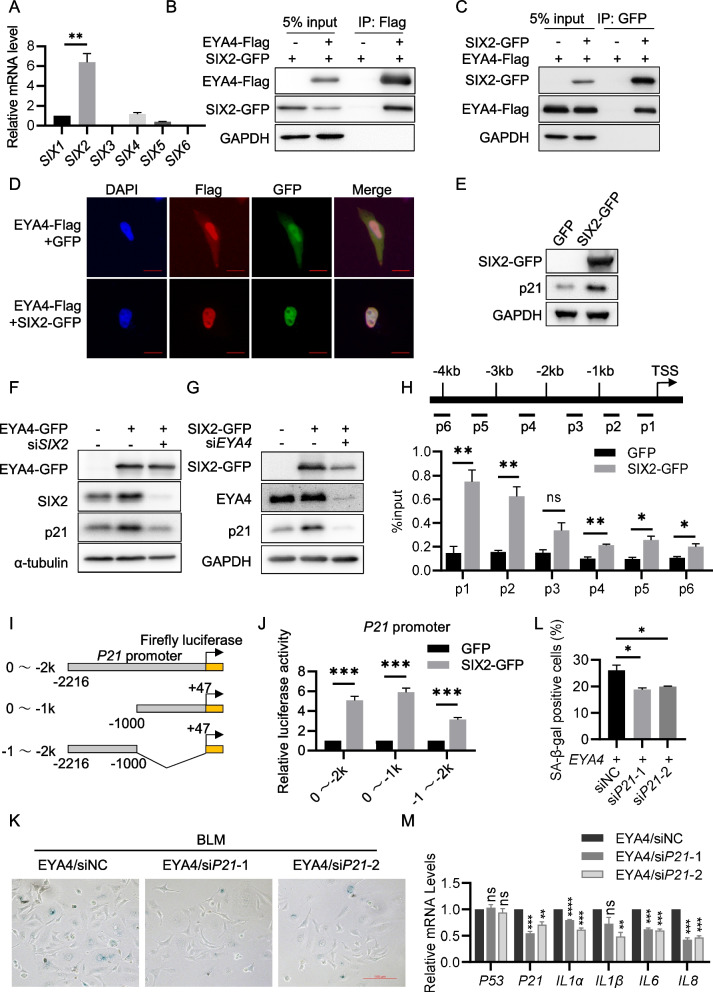
EYA4 interacts with SIX2 to promote *P21* transcription.** A** RT-qPCR analysis of endogenous SIX family proteins in HeLa cells. **B**, **C** Co-IP assays to determine the interaction between EYA4 and SIX2. HEK293T cells were transfected with EYA4-Flag and SIX2-GFP plasmids for 48 h and subjected to co-IP assay using Flag-tagged (**B**) or GFP-tagged (**C**) beads to assess the interaction between EYA4 and SIX2. **D** Immunofluorescence assay was performed to determine the subcellular localization of EYA4 (red) in HeLa cells. HeLa cells were transfected with EYA4-Flag and SIX2-GFP plasmids (GFP plasmid as control) for 48 h and subjected to immunofluorescence. Scale bars: 10 μm. **E** Immunoblot analysis of p21 expression level in HeLa cells. HeLa cells were transfected with SIX2-GFP or GFP for 48 h and subjected to immunoblot assay. **F** Immunoblot analysis of p21, EYA4 and SIX2 in HeLa cells. HeLa cells were transfected with siNC or *siSIX2* for 24 h and then transfected with EYA4-GFP or GFP plasmids for 48 h, and subjected to immunoblot assay. The α-tubulin was used as internal control because the molecular weights of GAPDH and SIX2 are similar, and it was difficult to distinguish them on the same membrane. **G** Immunoblot analysis of p21, EYA4 and SIX2 in HeLa cells. HeLa cells were transfected with siNC or *siEYA4* for 24 h and then transfected with SIX2-GFP or GFP plasmids for 48 h, and subjected to immunoblot assay. **H** Schematic illustration of primers used for ChIP-PCR targeting the *P21* promoter region (top) and ChIP-PCR results showing SIX2 enrichment at *P21* promoter binding sites (bottom). HeLa cells were transfected with SIX2-GFP or GFP plasmids for 48 h and subjected to ChIP-PCR assay.** I** Schematic diagram of luciferase reporter plasmids containing various lengths of the *P21* promoter. **J** Dual-luciferase reporter assay to determine the transcription of *P21* in HeLa cells. HeLa cells were transfected with luciferase reporter plasmids containing truncated *P21* promoter and SIX2-GFP plasmids (GFP plasmids as control). **K** SA-β-gal staining of HeLa cells transfected with *EYA4* plasmid and siRNAs as indicated. Cells were treated with BLM for 72 h prior to staining. **L** Quantification of (K). **M** RT-qPCR analysis of mRNA levels in cells described in (**K**). All values are means ± SEM of three independent experiments. Two-tailed unpaired Student’s t-test was used to determine the statistical significance (* *p* < 0.05, ** p < 0.01, *** p < 0.001)

According to previous studies, EYA family proteins lack DNA-binding ability, and SIX proteins are the well-known partners that interact with EYA proteins and promote EYA proteins into nucleus (Ohto et al. 1999). To determine whether EYA4 regulates p21 expression in a SIX protein-dependent manner, we first analyzed the expression correlation between SIX family members and EYA4 using the PEPIA website with the GTEx dataset. The results showed that the expression levels of *SIX1, SIX2* and *SIX4* were positively correlated with *EYA4,* whereas *SIX3, SIX5,* and *SIX6* were not (Supplementary Figure S2). We next compared the expression levels of *SIX1, SIX2* and *SIX4* across 12 tissues in which EYA4 is upregulated during aging (Fig. 1A) using GTEx dataset ("The Genotype-Tissue Expression (GTEx) project," 2013). It revealed that SIX2 exhibited the highest expression levels in most tissues among the three genes (Supplementary Figure S3). In addition, we detected the expression levels of SIX proteins and found SIX2 expression is the highest in HeLa cells (Fig. 5A). Therefore, we hypothesized that EYA4 may regulate p21 expression through its interaction with SIX2. To test this hypothesis, we first investigated whether EYA4 and SIX2 physically interact. It showed that overexpressed SIX2-GFP can be co-immunoprecipitated by exogenous EYA4-Flag, and reciprocally, EYA4-Flag can be co-immunoprecipitated by SIX2-GFP (Fig. 5B-C). In addition, overexpressed EYA4-Flag successfully pulled down endogenous SIX2 (Supplementary Figure S4A), further confirming their interaction. To determine which domain of EYA4 mediates binding to SIX2, we generated truncation constructs of NTD and ED for Co-IP assays. Our results demonstrated that the ED domain is primarily responsible for binding to SIX2 (Supplementary Figure S4B). Interestingly, however, the ED domain alone was insufficient to activate p21 expression (Supplementary Figure S4C), suggesting that the NTD is also required, likely due to its transcriptional activation function.

We next investigated how EYA4 activates p21 in cooperation with SIX2. Immunofluorescence analysis revealed that overexpression of SIX2 promoted the nuclear translocation of EYA4 (Fig. 5D). Conversely, SIX2 knockdown reduced the nuclear localization of EYA4 (Supplementary Figure S5). Together, these findings indicate that the interaction between SIX2 and EYA4 facilitates the nuclear translocation of EYA4. We then overexpressed SIX2 in HeLa cells and found p21 expression was also upregulated (Fig. 5E). These results suggest that EYA4 and SIX2 may work together to promote p21 expression. Further experiments showed that knocking down *SIX2* abolished the promoting effect of EYA4, and similarly, silencing *EYA4* negated the effect of SIX2 on p21 expression (Fig. 5F-G). This supports the idea that EYA4 and SIX2 cooperate in the regulation of p21 expression.

As mentioned above, EYA4 does not have DNA-binding capability, leading us to hypothesize that SIX2 binds to EYA4 and recruits it to promoter region of *P21*, thereby promoting *P21* transcription. To test this hypothesis, we performed the ChIP-qPCR experiment and found that SIX2 bound to the region approximately 1 kb upstream of the transcription start site (TSS) (Fig. 5H). Dual-luciferase reporter assays further confirmed that SIX2 binding within the 1 kb region promoted *P21* transcription (Fig. 5I-J). Subsequently, we simultaneously overexpressed EYA4 and knocked down *P21* in BLM induced senescent HeLa cells, followed by β-galactosidase staining and RT-qPCR analysis of SASP factors. The results showed that both the proportion of β-gal-positive cells and the expression of SASP factors were reduced upon *P21* knockdown (Fig. 5 K-M), indicating that EYA4-mediated senescence can be rescued by *P21* depletion.

Altogether, these findings indicate that the interaction between EYA4 and SIX2 promotes EYA4 nuclear translocation, facilitates its recruitment to the *P21* promoter, enhances *P21* transcription, and ultimately drives cellular senescence.

### EYA4 regulates p21 in a p53-dependent manner

**Fig. 6:**
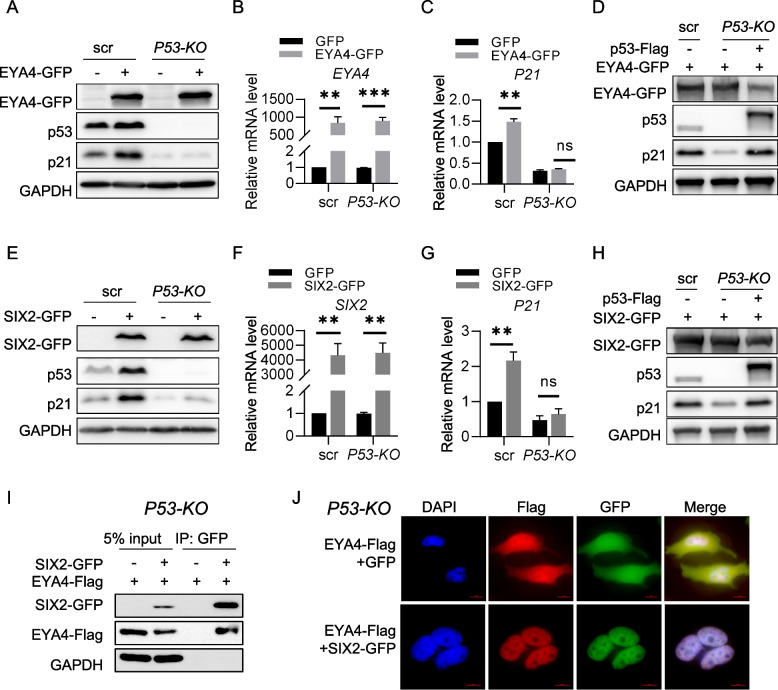
The regulation of p21 by EYA4 is governed by p53. **A** Immunoblot analysis of p21 in *P53-KO* HeLa cells. HeLa cells were transfected with EYA4-GFP or GFP for 48 h and subjected to immunoblot analysis. **B**,** C** RT-qPCR analysis of *EYA4* (**B**) and *P21* (**C**) mRNA levels in *P53-KO* HeLa cells described in (**A**). The y-axis in (**B**) is truncated to omit intermediate values due to the high fold change of EYA4 overexpression. **D** Immunoblot analysis of p21, p53 and EYA4 in *P53-KO* HeLa cells. Hela cells were transfected with EYA4-GFP plasmids for 48 h. The *P53-KO* HeLa cells were transfected with EYA4-GFP and p53-Flag plasmids (Flag plasmids as control) for 48 h. **E**–**H** The experiments were performed as described in (**A**-**D**), with SIX2 replacing EYA4. The y-axis in (**F**) is truncated to omit intermediate values due to the high fold change of SIX2 overexpression. **I** Co-IP assays to determine the interaction between EYA4 and SIX2 in *P53-KO* HeLa cells. *P53-KO* HeLa cells were transfected with EYA4-Flag and SIX2-GFP plasmids for 48 h and subjected to co-IP assay using Flag-tagged or GFP-tagged beads. **J** Immunofluorescence assay was performed to determine the subcellular localization of EYA4 (red) in *P53-KO* HeLa cells. *P53-KO* HeLa cells were transfected with EYA4-Flag and SIX2-GFP plasmids (GFP plasmid as control) for 48 h and subjected to immunofluorescence. Scale bars: 10 μm. All values are means ± SEM of three independent experiments. Two-tailed unpaired student’s t-test was used to determine the statistical significance (* *p* < 0.05, ** p < 0.01, *** p < 0.001)

Most previous studies have shown that p53 is a key and direct regulator of p21, with p53 binding directly to the *P21* promoter to activate its transcription (el-Deiry et al. 1994, 1993). However, there are also reports indicating that p21 can be activated in a p53-independent manner (Galanos et al. 2016). In this study, we found that SIX2 directly binds to the *P21* promoter and activates its transcription. To determine whether EYA4-mediated regulation of p21 depends on p53, we constructed *P53*-knockout (KO) HeLa cells, in which p21 expression was significantly reduced to undetectable level (Fig. 6A). We observed that EYA4 overexpression did not increase p21 expression in *P53-KO* cells (Fig. 6A-C). However, when p53 was rescued in these KO cells, p21 expression was restored to levels similar to those in *P53*-wild-type (WT) cells (Fig. 6D). In addition, the mRNA levels of *P21* and SASP factors were decreased significantly after *EYA4* knockdown in p53 rescued *P53-KO* cells (Supplementary Figure S6). We conducted the same experiment by overexpressing SIX2, and the results showed that SIX2 overexpression did not increase p21 expression in *P53-KO* cells either (Fig. 6F-I). These results suggest that the regulation of p21 by EYA4-SIX2 is significantly influenced by p53.

To explore whether p53 affects the EYA4–SIX2 interaction, we examined whether p53 is incorporated into this complex. Co-IP assays showed that neither EYA4 nor SIX2 was able to pull down p53, whereas the positive control Mdm2 successfully co-immunoprecipitated p53 (Supplementary Figure S7A). In addition, Co-IP results in *P53-KO* cells show that EYA4 also binds to SIX2 in the absence of p53 (Fig. 6I). These results indicate that p53 does not directly influence the interaction between EYA4 and SIX2. Moreover, the nuclear translocation of EYA4 was also enhanced by SIX2 in *P53-KO* HeLa cells, similar to that observed in wild type HeLa cells (Fig. 6J vs Fig. 5D). To further address the role of p53 in the EYA4–SIX2 axis, we performed ChIP-qPCR in *P53* knockout HeLa cells. The results showed that SIX2 binds to *P21* promoter in *P53-KO* cells with no obvious difference compared with *P53* wild-type cells (Supplementary Figure S7B vs Fig. 5H), suggesting that p53 does not directly affect the binding of the EYA4–SIX2 complex to the *P21* promoter. Moreover, to investigate whether the *P21* promoter remains responsive, we treated *P53-KO* or control cells with AKT inhibitor perifosine, which can activate p21 in a p53-independent way (Farhan et al. 2017). The result shows that AKT inhibitor successfully induced p21 upregulation in both cells (Supplementary Figure S7C). Together, these results reveals that *P21* promoter remains accessible and responsive in *P53-KO* cells, although the precise mechanisms by which p53 affects this regulation requires further investigation.

## Discussion

Aging is a multifactorial process driven by complex changes in gene expression. Increasing evidence has highlighted the dynamic regulation of gene expression during aging, especially the genes that are upregulated in senescent cells (Ou and Schumacher 2018; Roger et al. 2021; Torres et al. 2024). In this study, we observed a significant upregulation of EYA4 expression in aging individuals, as well as in replicative and stress-induced cellular senescence models, suggesting that EYA4 may be a key regulator of the aging process (Fig. 1). Our subsequent experiments demonstrated that knockdown of *EYA4* effectively delayed both replicative and stress-induced cellular senescence (Figs. 2–3). Hence, it seems that EYA4 elevation in aging tissues promotes the aging progress, mechanically by activating the expression of p21 (Figs. 4–5). The expression level of p16 did not changed after *EYA4* knockdown or overexpression (Fig. 4). It is reasonable because it has been reported that although p21 and p16 are two major regulators and markers for cell senescence, they are activated in distinct cell populations during aging (Wang et al. 2021). It was reported that p21 is primarily induced downstream of p53 in response to acute DNA damage and mediates an initial cell cycle arrest during early stage of senescence, whereas p16 is more frequently associated with stable and irreversible late senescent state (Scanlan et al. 2024). The selective upregulation of p21 by the EYA4–SIX2 axis therefore raises another possibility that this complex preferentially regulates early-stage senescence rather than late-stage senescence, potentially through cooperation with p53 at the *P21* promoter. However, this hypothesis requires further experimental validation, particularly given that cellular senescence is now recognized as a heterogeneous and dynamic process rather than a uniform endpoint state (Gorgoulis et al. 2019). As a cyclin-dependent kinase inhibitor, p21 inhibits the activity of CDKs (Graña et al. 1998; Narasimha et al. 2014; Sherr and Roberts 1995; Stevaux and Dyson 2002). Persistent activation of p21 induces cell cycle arrest and promotes the onset of cellular senescence (Guo et al. 2009; Stein et al. 1999). The high expression of p21 has been related to many kinds of age-related diseases, including metabolic disorders and cardiovascular diseases (Yan et al. 2024). There are three types of senescence that associated with p21 pathway, including replicative senescence, DNA damage induced senescence, and developmentally programmed aging (Yan et al. 2024). Here, we found EYA4 is upregulated during aging in many tissues, and promotes senescence by enhancing *P21* transcription in the first two types of senescence, suggesting EYA4 is a crucial regulator of aging and opening up potential therapeutic targets in delaying aging and age-related diseases. Given that EYA family is a critical regulator during mammalian organogenesis (Li et al. 2003), such as eye development (Pignoni et al. 1997), it is possible that EYA4-p21 axis also regulates developmentally programmed aging, which needs further study.

The EYA4 protein not only plays a crucial role in organogenesis development, but also regulates tumorigenesis, including both tumor-promoting and tumor-suppressing effects in different cancer types (de la Peña Avalos et al. 2023; Mo et al. 2018, 2016; Nelson et al. 2024; Zhu et al. 2019). The EYA4 dephosphorylates different kinds of substrates to inhibit or promote tumor progression. It dephosphorylates and activates PLK1 to promote cell proliferation and breast cancer progression (de la Peña Avalos et al. 2023; Nelson et al. 2024), and dephosphorylates β-catenin (Mo et al. 2016; Zhu et al. 2019) or IκBα (Mo et al. 2018) to suppress multiple cancers. In this study, we uncovered a new function of EYA4 to promote cellular senescence independent of its phosphatase activity, but depending on its transactivation activity. We observed that EYA4 interacts with transcription factor SIX2 to promote the expression of p21, providing valuable insights on the molecular regulation of cellular senescence. EYA4 does not have DNA-binding activity and therefore requires SIX2 to help it enter the nucleus and bind to DNA, while SIX2 needs EYA4 to enhance its transcriptional activation of *P21*. The two proteins are mutually dependent, and neither can function without the other. This finding is consistent with previous reports that members of the SIX family require interactions with members of the EYA family to activate gene transcription during development (Creed et al. 2020; Hirose et al. 2010; Ohto et al. 1999). However, there are still problems. There are six members in the SIX family. Previous studies have demonstrated that EYA proteins can interact with multiple SIX family members rather than being restricted to a single partner. For example, biochemical and developmental studies have shown that SIX2, SIX3, SIX4 and SIX5 regulate the intracellular distribution of EYA1, EYA2, EYA3 proteins through physical association (Ohto et al. 1999). These findings suggest that the specificity of EYA–SIX pairing may depend on tissue type, developmental stage, and relative expression levels of individual SIX proteins. It is possible that EYA4 will also interact with other SIX family members to regulate senescence-associated gene expression in different contexts. Further investigations into the interactions between EYA4 and other SIX proteins could uncover alternative regulatory pathways, highlighting the complexity of cellular senescence regulation. For instance, it would be interesting to explore whether EYA4-SIX1 interactions also promote p21 expression or contribute to senescence through other mechanisms, such as the regulation of additional senescence-associated genes. Another limitation of this study is the absence of endogenous co-immunoprecipitation or in vitro binding assays between EYA4 and SIX2, mainly due to the limited efficiency of currently available antibodies for immunoprecipitation and the technical difficulty of purifying these proteins in sufficient amounts. Nevertheless, previous structural studies of other EYA–SIX family members, such as the EYA2–SIX1 complex (Patrick et al. 2013), have demonstrated that these proteins interact directly through conserved domains. Given the high sequence and structural homology among EYA family members, it is therefore highly plausible that EYA4 also interacts directly with SIX2 via similar conserved motifs.

*P53* is a well-established tumor suppressor and regulator of cellular senescence, known for its ability to induce p21 expression, thereby promoting cell cycle arrest and senescence (Engeland 2022). In our study, we observed that upon knockdown of *P53*, the ability of EYA4 and SIX2 to activate p21 expression was abolished (Fig. 6), whereas supplementing p53 rescued the expression of p21 to normal level. It seems that the EYA4-SIX2 complex collaborates with p53 to regulate p21 expression. The p53 protein itself is a transcription factor that regulates a wide range of genes involved in cell cycle control, DNA repair, and apoptosis, and it is frequently involved in stress-induced senescence (Fischer 2017; Kern et al. 1991; Serrano et al. 1997; Xue et al. 2007). The mechanism by which EYA4-SIX2 interacts with p53 to regulate p21 expression remains to be fully elucidated. According to our results, SIX2 binds within 1 kb upstream of TSS of the *P21* promoter, while p53 binds 2.4 kb upstream of TSS (el-Deiry et al. 1993). It is plausible to speculate that p53 might be the determining factor for transcriptional initiation, whereas the EYA4–SIX2 complex functions as a co-regulatory module to fine-tune p21 expression. Further studies are needed to investigate the precise molecular interactions between EYA4, p53, and other senescence-related proteins to fully understand how this pathway contributes to the aging process.

## Supplementary Information

Supplementary Material 1.

Supplementary Material 2.

Supplementary Material 3.

## Data Availability

The original contributions presented in the study are included in the article/supplementary material.
