# Supplementary material for: EYA4 promotes cellular senescence by enhancing P21 transcription through interaction with SIX2
Source: Adv Biotechnol (Singap). 2026 Apr 17;4(2):17. doi: 10.1007/s44307-026-00109-8 (PMC13087065; doi:10.1007/s44307-026-00109-8)
Supplement: Supplementary file 3 — Supplementary Material 3. [file 44307_2026_109_MOESM3_ESM.pdf]

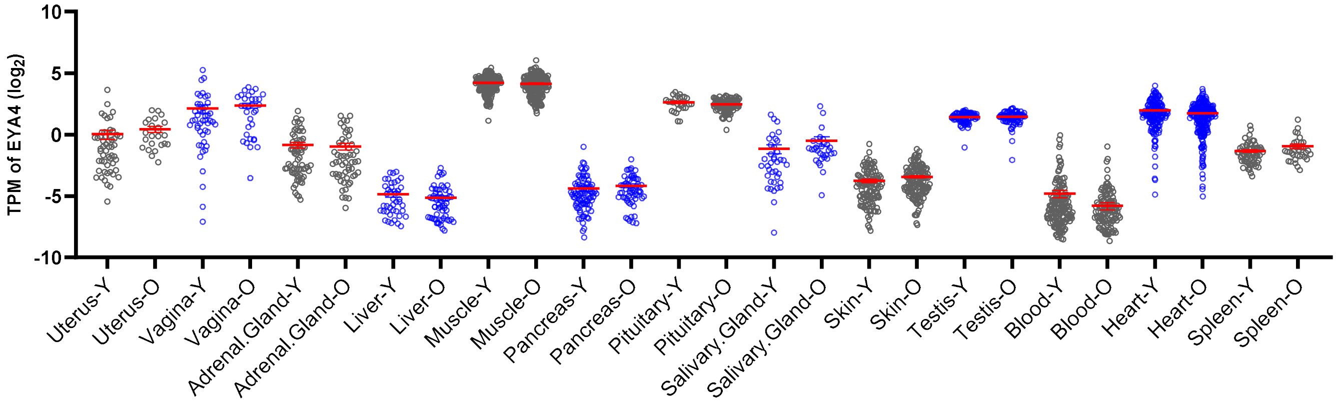

Figure S1 Expression levels of *EYA4* in 13 tissues from young (20–49 years) and elderly (≥60 years) individuals based on RNA-seq data obtained from the GTEx database. The sample size was between 30 and 223 across tissues. The y-axis was $\log_2$-transformed to improve visualization of differences across a wide range.

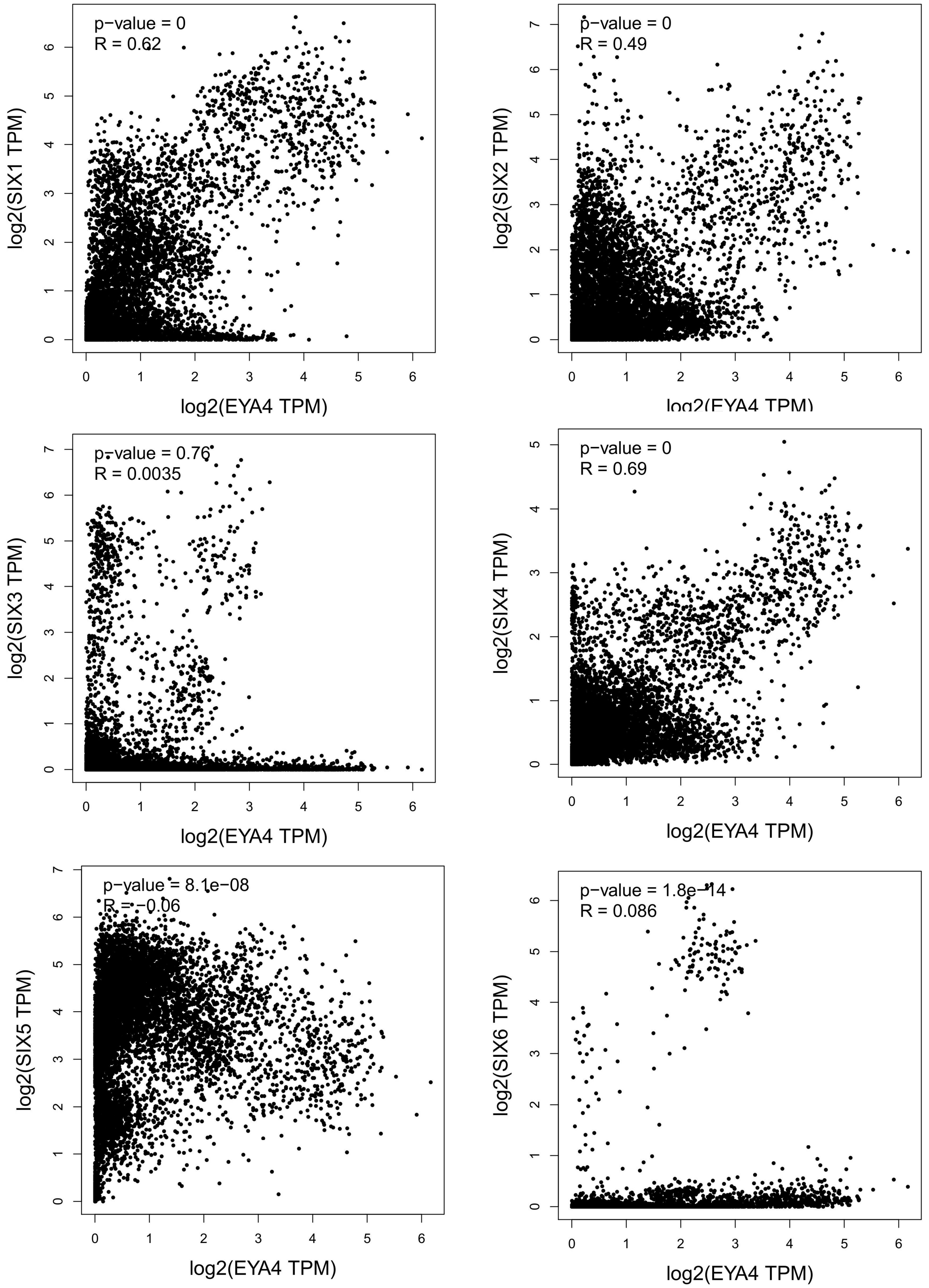

Figure S2 Correlation between *SIX* family gene expression and *EYA4*. Pearson correlation analysis of gene expression between *SIX* family members (*SIX1–SIX6*) and *EYA4* was performed in PEPIA website using the GTEx dataset (TPM), including all tissue types. Each panel shows the correlation between indicated *SIX* member and *EYA4*。

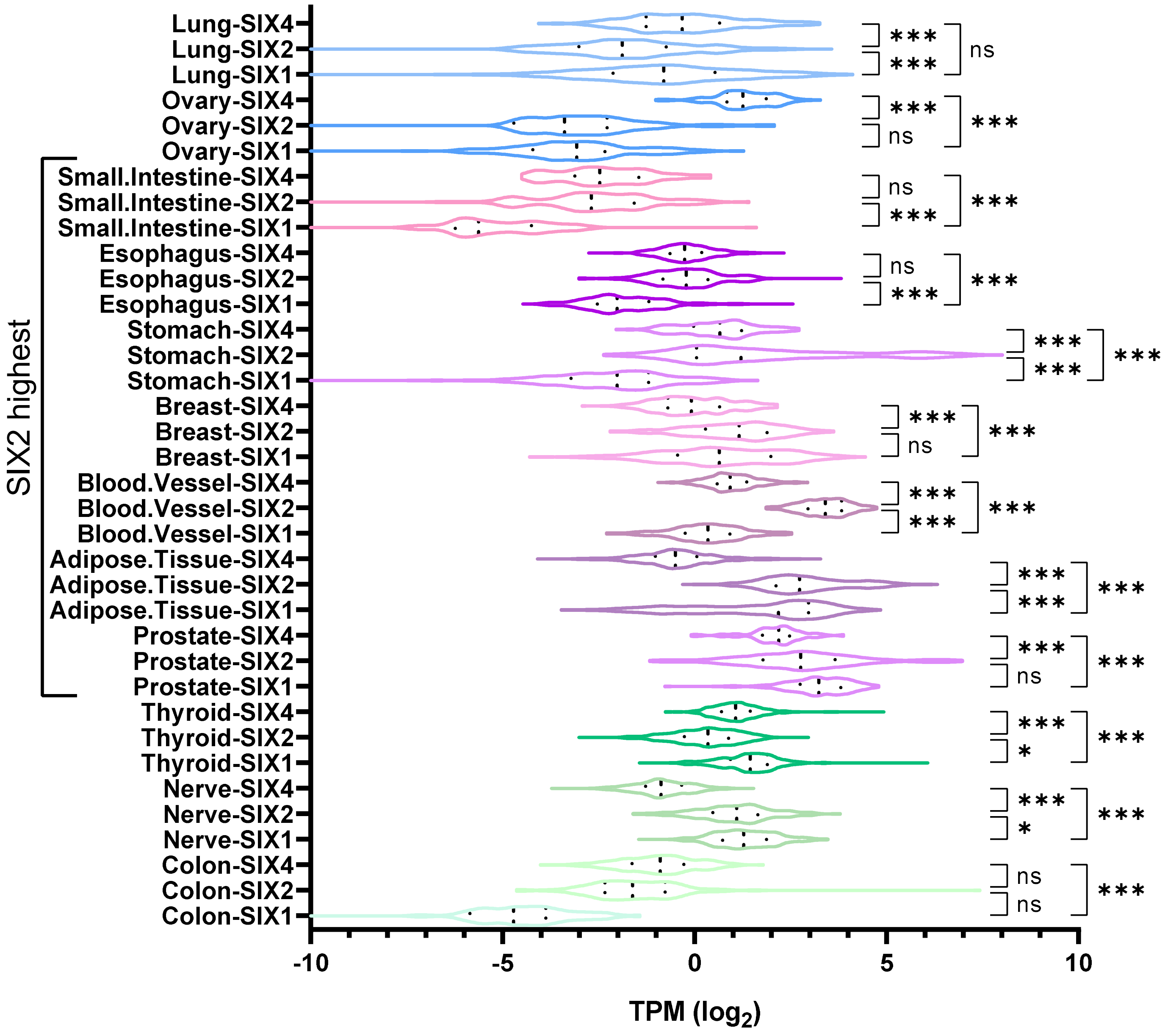

Figure S3 Expression levels of *SIX1, SIX2* and *SIX4* in human tissues。The expression levels of three genes were compared in tissues showed in Figure 1A based on RNA-seq data obtained from the GTEx database. The sample size was between 99 and 572 across tissues. The x-axis was log_2-transformed to improve visualization of differences across a wide range.

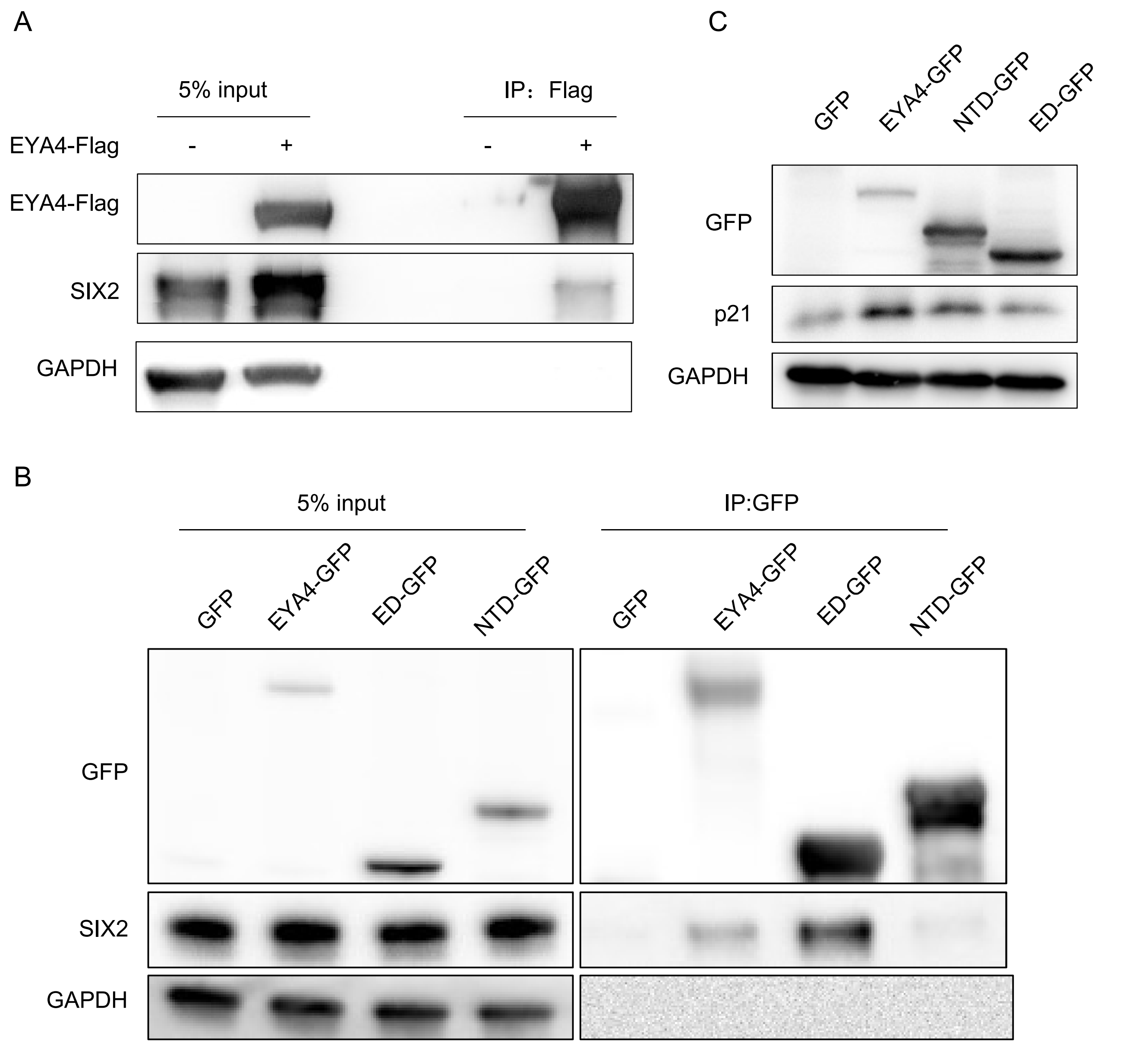

Figure S4 Co-IP assays between EYA4 and SIX2. (A) Co-IP using anti-Flag beads to examine the interaction between exogenous EYA4-Flag and endogenous SIX2. (B) Co-IP analysis of truncated EYA4 with endogenous SIX2. (C) Immunoblot analysis showing p21 expression after transfection with full-length or truncated *EYA4* plasmids.

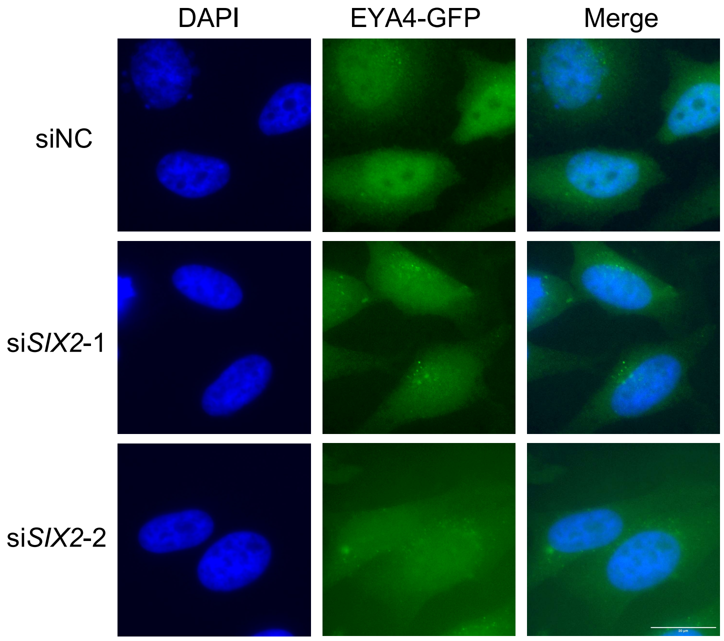

Figure S5 Representative immunofluorescence images showing the subcellular localization of EYA4 in SIX2-deficient HeLa cells.

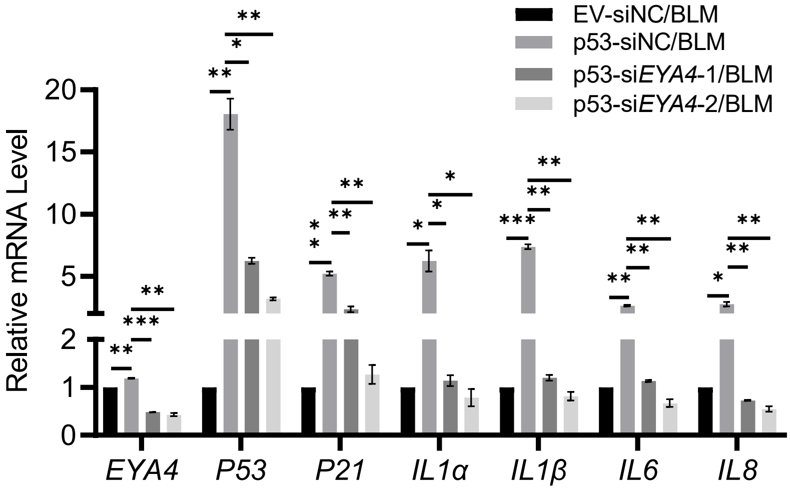

Figure S6 P53 overexpression rescues EYA4 function in *P53-KO* cells. P53 was overexpressed in *P53-KO* HeLa cells while *EYA4* was simultaneously knocked down. Cells were treated with BLM for 72 h, followed by qRT-PCR analysis.

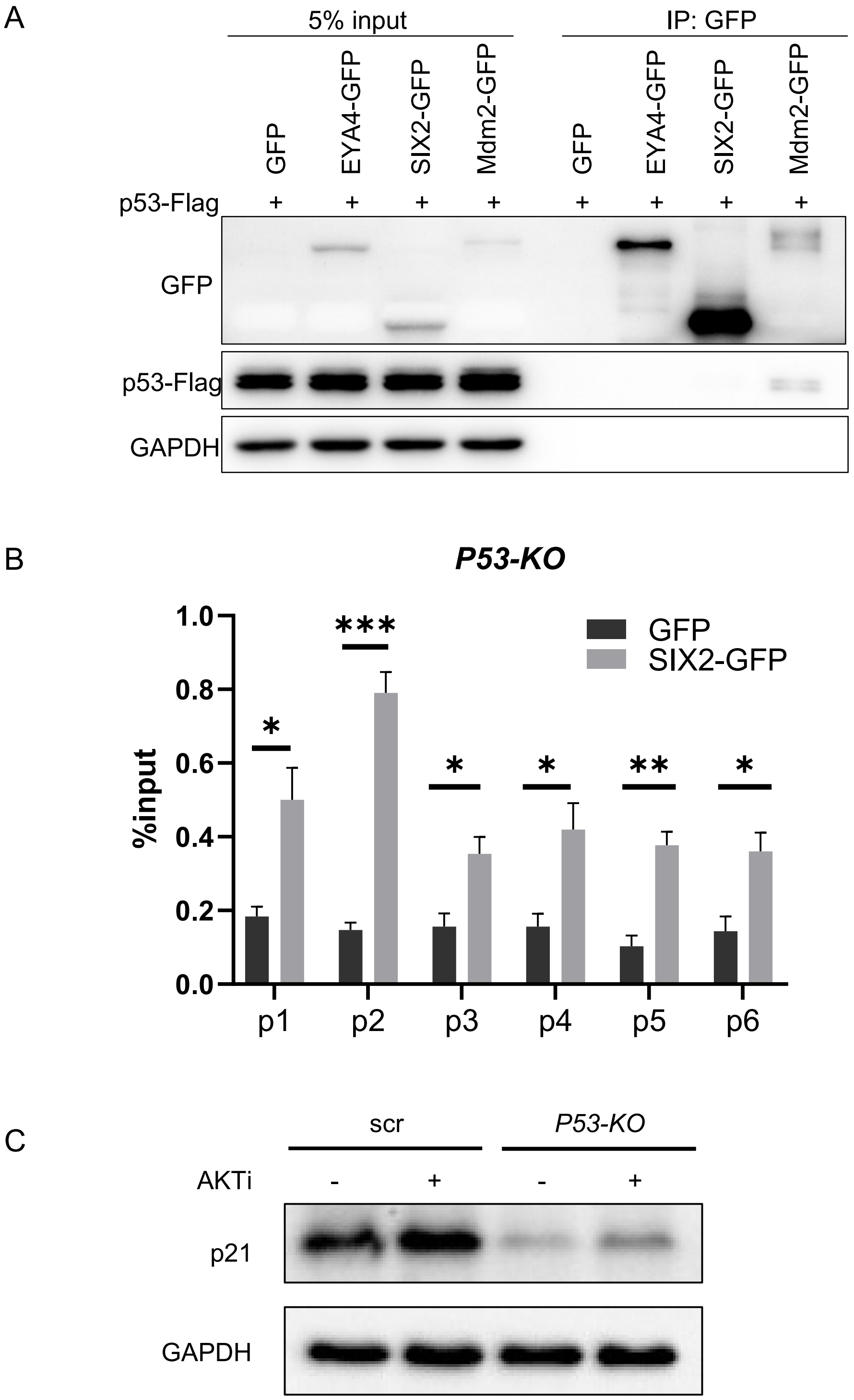

Figure S7 P53 is not required for EYA4-SIX2 interaction or binding to *P21* promoter. (A) Co-IP showed no interactions between EYA4/SIX2 and p53. (B) ChIP-PCR data showing SIX2 enrichment at *P21* promoter in *P53-KO* HeLa cells.(C) Immunoblot analysis of p21 protein level with the treatment of AKT inhibitor Perifosine.
